# Relationship between Thyroid Hormone Levels and Crime Type: A Controlled Study in Prisoners

**DOI:** 10.1155/2020/9172134

**Published:** 2020-03-09

**Authors:** Hasan Acar, Ayse Ulgen

**Affiliations:** Girne American University Medical Faculty, Karmi, Cyprus

## Abstract

Various factors cause aggression, which can be related to imbalance of T3 and T4 hormones, which can act as neurotransmitters and are reported to be elevated during aggression. This indicates changes in the hypothalamic-pituitary-thyroid axis that cause long-term changes in aggressive behaviour, especially in criminals. Moreover, mental and behavioural disorders possibly occur in individuals with impairment in thyroid hormone balance. The main rationale for this study was to asses if high T3, high T4, and low TSH hormones may have an effect on aggression-related crime tendency. Furthermore, the study aimed to measure levels of thyroid hormones in prisoners and to examine relationships of the hormone levels with crime rates. Our study was conducted in Ankara Sincan Closed Prisons. The study group consisted of 208 male volunteers who were imprisoned and the control group included 82 male volunteers who were not imprisoned. Prisoners in the study group were divided into two groups: those who committed aggression-related crime (Group A, *n* = 96) and prisoners convicted of other crimes (Group B, *n* = 112). Pulse rates, T3, T4, and thyroid-stimulating hormone (TSH) levels, and theT3/T4 ratio were measured in these prisoners. Data were analysed using the Wilcoxon rank sum test and chi-square Fisher's exact test to test for any statistically significant differences. Results showed that toxic goitre rates, T3 and T4 values, and pulse rates were significantly higher in Group A than in the control group. Significant increase in T3 and T4 levels and the presence of toxic goitre were associated with aggression-related crime. These examinations should be performed on prisoners in general, especially those convicted of violent crimes. Additional rehabilitation and research programs should also be developed for such patients.

## 1. Introduction

Genetic causes, hormones, environmental factors, and some diseases may contribute to aggression in individuals. In a consortium of 12 projects supported by the European Union where new research on aggression was conducted in a large study group consisting of 18,988 individuals, gene-based analyses were performed to investigate the genomic structure underlying aggressive behaviour in children. In this study, it was stated that functional variation in arginine vasopressin receptors may be responsible for higher levels of aggressive behaviour [1]. In another study conducted by Malik et al., oxytocin and oxytocin receptor gene variants were examined in early childhood aggression. OXTR SNPs rs6770632 variants in girls and rs1042778 variants in boys were associated with excessive and persistent aggressive behaviour [2].

Studies conducted thus far have reported that some biomarkers play a role in the emergence of aggressive behaviour. Harro and Oreland demonstrated that high levels of monoamine oxidases (MAO-A and MAO-B) lead to a labile personality structure that may increase aggression [3]. Foster and Spitz showed that changes in serum albumin, calcium, chloride, haemoglobin, thyroxin, mean corpuscular haemoglobin concentration, mean corpuscular volume, and red blood cell count were associated with aggression [4]. Coccaro et al. demonstrated the association of elevated blood serotonin with aggression [5]. Simon et al. have implicated elevated serum testosterone level in aggression [6–8]. Carbonell et al. demonstrated a correlation between aggression and elevated serum adrenocorticotropic hormone (ACTH) [9]. Handa et al. showed that elevated serum oestrogen was related to aggressive behaviour [10, 11].

Immunohistochemical studies conducted by Dratman and Gordon have shown that T3 is concentrated in the brain in the noradrenergic centres and acts as a neurotransmitter [12]. The study conducted by Mainardi et al. reported that blood T3 and T4 levels were high in dominant aggressive mice [13]. The role of thyroid hormones in the regulation of aggression in dogs was identified by Dodman et al. [14]. A study conducted by Eklund et al. evaluated the relationship between behavioural disorders at early ages and biochemical variables in adulthood and their importance in terms of early crime and lifetime violent behaviours. The results showed a significant combined risk level pattern of low MAO activity and high T3 level in violent offenders with early behavioural risk patterns [15].

In a forensic psychiatric study conducted by Stalenheim et al., T3 and platelet MAO activity were measured in criminal recidivist, nonrecidivist, and control groups. In the criminal recidivist group, where individuals had psychopathy and aggression related to antisocial personality disorder (APD) according to the “Karolinska Scale of Personality,” T3 level and MAO activity were higher than those in the nonrecidivist and control groups. These results suggest that changes in the hypothalamic-pituitary-thyroid axis can cause chronic changes in this group of individuals [16].

Individuals with mental and behavioural disorders may have impaired thyroid hormone level balance. In a controlled study conducted by Sinai et al., thyroid hormone levels in 57 patients with borderline bipolar disorder (BPD) were compared with those of a control group. The serum T3 levels were found to be significantly higher in the study group than in the control group [17]. In a study conducted by Baz et al., serum thyroid hormone and thyroid-stimulating hormone (TSH) levels were measured in 30 patients with attention-deficit hyperactivity disorder (ADHD). All patients had high T3 and low T4 levels, and half of the patients had high TSH, while the other half of them had normal levels [18]. A study conducted by Radhakrishnan et al. showed impairment in thyroid hormone balance in many mental illnesses ranging from BPD to schizophrenia [19]. In a study conducted by Evrensel et al., T3 and T4 values were measured in criminal and noncriminals with antisocial APD. Mean free T3 levels and aggression scores were significantly higher in the criminal group than in the noncriminal group [20].

Goitre is a common endemic disease in the Mediterranean region. Studies have reported that one out of every five people in Turkey has goitre and approximately 0.1% of them have toxic goitre [21, 22]. Therefore, there is a possibility of serious mental and social problems due to toxic goitre. The main rationale for this study was to asses if high T3, high T4, and low TSH hormones may have an effect on aggression-related crime tendency. Therefore, we investigated the association of thyroid hormone levels, pulse rate, TSH, T3/T4 ratio, and presence of toxic goitre with crime type in prisoners.

## 2. Materials and Methods

All procedures performed in this study were in accordance with the ethical standards of the institutional and/or national research committee and the 1964 Helsinki Declaration and its later amendments or comparable ethical standards. No animal studies were carried out by the authors for this study. All volunteers who participated in the study were informed about the aims of the study and the possible benefits of the study to the scientific community and themselves, and their consent was obtained.

Our study was conducted in Ankara Sincan Closed Prisons. The study group consisted of 208 male volunteers who were imprisoned and the control group had 82 male volunteers who were not imprisoned (Group cont.). Only male prisoners were included in the study in order to restrain the effects of hormonal changes due to menstrual periods. Blood samples were collected from individuals of both groups, and T3, T4, and TSH levels and T3/T4 ratio were measured. Toxic goitre examinations and pulse rate measurements were also performed. Then, the prisoners in the study group were further divided into two subgroups: Group A (*n* = 96), consisting of prisoners convicted of aggression-related crimes including murder, looting, intentional assault, assault, violence in institutions, penetrating tool violence, torture, torment, forced rape, sexual assault, and damage to property, and Group B (*n* = 112), consisting of prisoners convicted of crimes of other types not related to aggression, including smuggling, theft, fraud, drug trafficking, misleading tender, embezzlement, and bribe.

After the results were compared between the groups and evaluated statistically, toxic goitre cases (Group D) identified in Group A were subgrouped (Group C). Pulse rate, T3, T4, and TSH levels and T3/T4 ratio were compared between Group B and Group cont. and analysed statistically. Repeated crime rates in Group A and Group B were compared and evaluated statistically.

Statistical analyses were performed using IBM SPSS Statistics 23 and R. Package. Pulse rate, TSH, T3, and T4, levels, T3/T4 ratio, and repeated crime rates were calculated and compared among Groups A, B, C, and D, and the data were recorded as mean, standard deviation. Additionally, we compared Group cont. vs. Group A, Group cont. vs. Group B, Group A vs. Group B, Group cont. vs. Group D, Group C vs. Group B, and Group cont. vs. Group C for these variables. We checked the data for normality and as the data were nonnormally distributed, we used the Mann–Whitney U nonparametric test for comparing pulse rate, TSH, T3, and T4 levels, and T3/T4 ratio, with *p* ≤ 0.01 indicating significance. For toxic goitre cases, we compared Group A vs. Group B and Group A vs. Group cont. using a chi-square Fisher exact test.

## 3. Results

The mean age (standard deviation) and distribution of crime among the individuals of the study and control groups are shown in Table 1.

When the mean age of the patients in Group A, Group B, and Group cont. were compared, no statistically significant differences were found (Table 1).

The results of presence of toxic goitre; mean pulse rate; mean T3, T4, and TSH levels; and mean T3/T4 ratio (standard deviations) of individuals in the study and control groups are shown in (Table 2).

Table 3 shows the mean values (standard deviations) of serum T3, T4 and TSH levels, mean pulse rate, and mean T3/T4 ratio of patients with toxic goitre (Group D) in the study group and those in the control group.

Table 4 shows a comparison of the mean values (standard deviations) of pulse rates, T3, T4 and TSH levels, and T3/T4 ratio measured after excluding prisoners with toxic goitre in Group A (Group C) with those of Group B prisoners and the control individuals.

The results for repeated crime rates in Group A and Group B are shown in Table 5.

Table 6 shows the Wilcoxon rank sum test results comparing mean values of pulse rate, TSH, T3, and T4 levels, andT3/T4 ratio, with *p* ≤ 0.05 indicating significance (presented in bold in the table). When comparing Group A and Group cont., all variables showed significant differences. When comparing Group B and Group cont., only pulse rate and TSH levels showed significance. There were no significant differences between T3, T4, and T3/T4 levels. When Groups A and B were compared, pulse rate, T3, and T4 showed significant difference, but there was no significant difference in TSH levels between Groups A and B. When control and Group D were compared, all values showed significant differences. When Groups C and B were compared, there were no significant differences for any of the variables. When Group C and Group cont. were compared, all variables showed significant difference.

With chi-square Fisher's exact test, we obtained a *p*-value of 0.02 when comparing Groups A and B and a *p*-value of 0.06 when comparing Group A and the control group. Therefore, there was a significant difference between Group A and control group for toxic goitre cases at the *p* ≤ 0.05 significance level but not for Group B and the control group.

## 4. Discussion

When the study group was subdivided into aggression-related offenders (Group A) and offenders convicted of other crimes (Group B), toxic goitre rates, T3, T4, pulse rates, and T3/T4 ratio values were found to be significantly higher in Group A than in the control group. In Group B prisoners, only pulse rates were found to be statistically higher than those in controls, and TSH values were found be lower than those in the control group (Tables 2 and 6). According to these results, presence of toxic goitre and high T3, T4, and T3/T4 ratio values were associated with increased tendency for aggression-related crime but did not show association with nonaggression crime. TSH values were found to be significantly lower and pulse rates were significantly higher in all study groups than in controls (Tables 4 and 6). According to these results, when other variables such as heart disease and anaemia are ruled out, an increase in pulse rates may indicate a crime-prone personality. Although the difference in pulse rates between Group B and control group was statistically significant, the difference in thyroid hormone levels between these groups was not significant. According to these findings, an increased pulse rate is associated with violent crime independently of thyroid hormone values (Table 6).

In our study, although there was an increase in both T3 and T4 values in individuals with toxic goitre in Group D, T3/T4 ratio was found to be significantly higher than that in the control group (Table 3 and 6). Stalenheim et al. measured free T3 and free T4 values in 66 normal controls and 61 nonpsychotic male subjects in forensic psychiatric examinations and followed up the subjects for 15 years. They stated that high T3 values significantly increased the tendency to commit psychopathy and crime and that high T3 and low free T4 values could be accepted as biological markers [23].

Soderstrom et al. conducted a study with a group of 37 nondependent aggressive criminals. They measured T3 and T4 values in serum and psychopathy rating in parallel with an increase in peripheral deiodination [24].

Although Stalenheim et al. reported an increase in T3 values and T3/T4 ratio in patients with psychopathy [23, 24], according to the results of our study, T4 values were also significantly higher in Groups A, C, and D than in the control group (Table 6). The common feature of Groups A, C, and D is that they all committed crimes related to aggression. T4 values in Group B who committed crimes not related to aggression were found meaningless compared to the control group. Unlike the results of the studies of Stalenheim and Soderstrom, T4 hormone can also be used as a biomarker in people who commit aggression-related crime.

T3 and T4 values of those in Group C (after exclusion of toxic goitre cases) were compared with those of the control group. The differences were statistically significant (Tables 4 and 6). According to these results, high T3 and T4 values (below toxic values) are also associated with the tendency to commit aggression-related crimes.

Some studies state that hyperthyroidism increases psychiatric morbidity. In patients with toxic goitre, mental lability, aggression, delirium, and paranoid and manic psychoses can occur. In a study performed by Brandt et al., 2631 hyperthyroid patients were examined and followed up for 6 years and their results were compared with those of a nonhyperthyroid control group. Hyperthyroid patients were reported to experience significantly more psychiatric problems and receive more treatment before and after diagnosis compared to the controls, both before and after diagnosis [25]. Hyperthyroid patients may show aggression, especially in paranoid and manic psychoses. Despite treatment of toxic goitre in these patients, psychological disorders can persist for a long time [26]. In a study conducted by Brownlie et al., 18 cases of toxic goitre and psychosis were examined. Among these cases, 7 were manic, one was paranoid, and one had delirium-type psychosis. After diagnosis, antithyroid and antipsychotic treatment was initiated to patients and the psychotic state was completely improved in more than half of the patients over an 11-year follow-up period [27]. Similarly, cases of Hashimoto thyroiditis [28] and subacute thyroiditis [29] with psychotic episodes have been reported.

In these studies [25–27], the rates of those with toxic goitre who committed aggression-related offenses were not specified. In addition, studies investigating aggression-related offenders in cases with toxic goitre could not be found in the literature.

Higher crime rates related to aggression in patients with toxic goitre are important in terms of forensic psychiatry.

If results are obtained in similar studies, as with alcoholics or drug addicts, toxic goitre patients may be exempted from punishment.

In our study, although toxic goitre was found in 5.2 % of Group A prisoners, it was not found in Group B or Group cont. The differences were statistically significant (Table 2). According to official data (TÜİK), the number of detainees and convicts in Turkey in 2019 was 286 000 [30]. When calculated for the rates of toxic goitre in these prisoners, a significant proportion of those convicted of offenses related to aggression had toxic goitre [21, 22, 30].

Although Graves' disease is an autoimmune disease, it has been suggested that a stressful lifestyle can affect neuroendocrine and immune systems and converts simple goitre to diffuse or nodular toxic goitre [31]. In a study conducted by Eleftheriou et al., serum and pituitary TSH values were measured after fight at regular intervals in the study group of mice. TSH levels in the serum and the pituitary gland increased significantly and persisted for 2 days after the onset of aggression in the study group compared to that in the control group [32].

The results of these studies show that stressful environmental conditions may affect the hypothalamus-pituitary-thyroid axis, increase thyroid hormone levels, and lead to aggression. Thus, a vicious cycle may occur.

Many of the volunteer prisoners who participated in our study did not have a forensic psychiatric examination. Therefore, we do not know whether inmates with toxic goitre had a preconviction toxicity or whether a toxic goitre was caused by the stress of conviction. We also do not know whether they had psychiatric illness. In addition, we have not yet been able to follow the long-term results of toxic goitre treatment in prisoners in Group D. Therefore, we could not investigate how treatment affects aggressiveness in these patients.

Browlie et al. reported that in half of patients with toxic goitre and psychosis, the psychotic state completely improved as a result of antithyroid therapy [27]. Therefore, we propose that the effect of antithyroid therapy on the prevention of aggression-related crimes should be investigated, especially in toxic goitre cases causing manic-, paranoid-, and delirium-type psychoses with aggressive behaviours and in personality disorders related to aggressive behaviours.

In a study conducted by Alm et al., T3 and TSH values in 70 juvenile and 35 control subjects who had previously committed crimes were examined and followed up for 15 years. T3 values were found to be 3.8 times higher in the study group than in the noncriminal control group, and TSH values were not related to variables [33]. In our study, TSH values were found to be lower in all study groups than in the control group (Table 6). According to these results, low TSH values can be an important factor in crime evaluations.

Previous thyroid hormone levels were not known for our study prisoners, but the difference between repetitive crime rates in Group A and Group B was not statistically significant (Table 5). However, since we do not know the previous thyroid hormone values of the prisoners included in our study, we cannot fully evaluate the factors affecting this result. Apart from this, another limitation of our study is that for those patients in which toxic goitre is detected (Group D), long-term follow-up is not assessed, and aggressive personality changes and repeated crime rates are not revealed in prisoners. When these limitations are removed in future studies, the factors affecting the repeated crime rates will be more clearly revealed.

**Figure 1:**
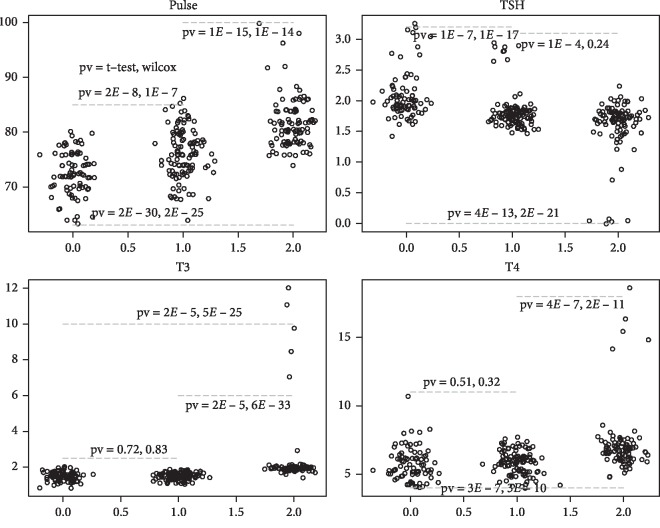
The clustering of the values for pulse rate, TSH, T3, and T4 for Group cont., Group B, and Group A, respectively.

Figure 1 shows the clustering of values for pulse rate, TSH, T3, and T4 for Group cont., Group B, and Group A, which shows how the three groups are separable. This is interesting because it implicates that it may be possible to predict whether someone has criminal tendency or not by using the above measurements.

## 5. Conclusions

According to the results of our study, toxic goitre and high levels of T3 and T4 hormones may increase the tendency for aggression-related crimes in humans. In this context, we believe that forensic psychiatric examinations can be performed in individuals with aggression, especially in offenders who commit crime related to aggression. Additionally, new treatment, rehabilitation, and research programs should be developed for these patients and these projects should be supported by national funds.

## Figures and Tables

**Table 1:** Mean age, standard deviation, and distribution of crime among individuals of the study and control groups.

Groups	Mean age	sd	Number of individuals
Group A	35.59	(±6.14)	96
Group B	35.15	(±5.84)	112
Group cont.	37.17	(±7.48)	82
Total			290

Group A: individuals convicted of crimes related to aggression. Group B: prisoners convicted of other crimes, not related to aggression.

**Table 2:** Presence of toxic goitre, mean pulse rate, mean T3, T4, and TSH levels, and T3/T4 ratio according to the group.

Groups	Toxic goitre *n*, %	Pulse rate Mean (sd)	T3 Mean (sd)	T4 Mean (sd)	TSH Mean (sd)	T3/T4 Mean (sd)
Group A	5, 5.2	81.64 (±4.66)	2.33 (±1.79)	7.14 (±2.21)	1.63 (±.40)	0.31 (±.09)
Group B		76.13 (±4.41)	1.50 (±.19)	5.86 (±.86)	1.81 (±.27)	0.26 (±.05)
Group cont.		72.54 (±4.00)	1.49 (±.25)	5.77 (±1.12)	2.08 (±.36)	0.27 (±.07)

Group A: individuals convicted of crimes related to aggression. Group B: prisoners convicted of other crimes, not related to aggression.

**Table 3:** Mean values (standard deviations) of serum T3, T4 and TSH levels, pulse rate, and T3/T4 ratio in patients with toxic goitre (Group D and those in the control group).

Groups	Pulse rate Mean (sd)	T3 Mean (sd)	T4 Mean (sd)	TSH Mean (sd)	T3/T4 Mean (sd)
Group D (*n* = 5)	95.60 (±3.58)	9.68 (±2.01)	15.95 (±1.67)	0.10 (±.04)	0.61 (±.14)
Group cont.	72.54 (±4.00)	1.49 (±.25)	5.77 (±1.12)	2.08 (±.36)	0.27 (±.07)

Group D: convicts with toxic goitre.

**Table 4:** Comparison of the mean values of pulse rates, T3, T4 and TSH levels, and T3/T4 ratio of individuals Group C with those of individuals in Group B and control group.

Groups	Pulse rate Mean (sd)	T3 Mean (sd)	T4 Mean (sd)	TSH Mean (sd)	T3/T4 Mean (sd)
Group C (*n* = 91)	80.87 (±3.29)	1.92 (±.15)	6.66 (±.70)	1.71 (±.19)	0.29 (±.04)
Group B	76.13 (±4.41)	1.50 (±.19)	5.86 (±.86)	1.81 (±.27)	0.26 (±.05)
Group cont.	72.54 (±4.00)	1.49 (±.25)	5.77 (±1.12)	2.08 (±.36)	0.27 (±.07)

Group C: remaining offenders after excluding those with toxic goitre from Group A. Group B: prisoners convicted of other crimes.

**Table 5:** Repeated crime rates in Group A and Group B.

Groups	Number of criminals	Repeated crime rates Mean (sd), %^*∗*^
Group A	96	2.38 (±1.05), 85.42
Group B	112	2.54 (±1.02), 83.04

Group A: prisoners convicted of crimes related to aggression. Group B: prisoners with other crimes, not related to aggression ^*∗*^Crimes committed at least two times or more.

**Table 6:** Wilcoxon rank sum test results for pulse rate, TSH, T3, and T4, and T3/T4 ratio.

Groups	Z (*p*-value)				
	Pulse rate	TSH	T3	T4	T3/T4
Group A vs. Group cont.	**−10.43 (<0.001)**	**−9.50 (<0.001)**	**−10.33 (<0.001)**	**−6.31 (<0.001)**	**−3.70 (<0.001)**
Group B vs. Group cont.	**−5.31 (<0.001)**	**−8.56 (<0.001)**	−0.22 (0.83)	−1.00 (0.32)	−0.54 (0.59)
Group A vs. Group B	**−7.73 (<0.001)**	−1.18 (0.24)	**−11.96 (<0.001)**	**−6.7 (<0.001)**	
Group D vs. Group cont.	**−3.77 (<0.001)**	**−3.74 (<0.001)**	**−3.74 (<0.001)**	**−3.74 (<0.001)**	**−3.74 (<0.001)**
Group C vs. Group B	−0.62 (0.53)	−0.62 (0.54)	0.62 (0.54)	0.62 (0.54)	
Group C vs. Group cont.	**−10.24 (<0.001)**	**−9.23 (<0.001)**	**−10.13 (<0.001)**	**−5.95 (<0.001)**	

Group A: prisoners convicted of crimes related to aggression. Group B: prisoners convicted of other crimes. Group C: remaining offenders after excluding toxic goitre cases from Group A. Group D: convicts with toxic goitre.

## Data Availability

The data used to support the findings of this study are available from the corresponding author upon request.
